# Normative Beliefs about Adolescent-to-Parent Violence: The Spanish Adaptations of the Beliefs about Child-to-Parent Abuse Questionnaire and the Abusive Behavior by Children-Indices

**DOI:** 10.3390/healthcare11202775

**Published:** 2023-10-20

**Authors:** Helena Cortina, Ana M. Martín

**Affiliations:** Departamento de Psicología Cognitiva, Social y Organizacional, Facultad de Psicología y Logopedia, Campus de Guajara, Universidad de La Laguna, 38200 San Cristóbal de La Laguna, Spain; hcortina@ull.edu.es

**Keywords:** adolescent-to-parent violence, BACPAQ, ABC-I, cultural differences, gender

## Abstract

Ascertaining the true prevalence of adolescent-to-parent violence (APV) is challenging because the measurement of APV in research is complex. There is no consensus on which behaviors constitute APV or how frequently they need to occur to be considered abusive. This study aimed to explore the normative beliefs about APV related to the perpetrator’s gender in a sample of Spanish parents, by developing Spanish adaptations of the BACPAQ and the ABC-I. The participants were 329 Spanish parents aged 19 to 81, and 77% were mothers. They answered the Spanish adaptation of the BACPAQ online after being contacted by university students using the snowball sampling technique. Results show that sons were judged more harshly than daughters; although, differences were statistically significant only for a few psychologically abusive behaviors. There was agreement with the original study on the abusive nature of most behaviors, especially regarding physical violence. Cultural differences were reflected in verbal, psychological, and economic violence, and Spanish parents used more stringent thresholds than Australians. Future research should tackle the difficulty of carrying out studies on APV using a single tool able to reflect normative beliefs about this type of domestic violence in different cultures.

## 1. Introduction

Adolescent-to-parent violence (APV) is a type of domestic violence that has gradually attracted media and research attention in different parts of the world because of its increase in reports. Since 2006, the Spanish Attorney General’s Office’s annual reports have repeatedly drawn attention to the disheartening increase in APV cases and to the failure of juvenile courts and reform authorities to tackle this situation in Spain. The 2020 report, for example, draws attention to a 16.07% increase from 2016 to 2019 in APV judicial cases, calling it “disheartening” [1] (p. 938). This increase, however, may be interpreted in different ways depending on the country. In Spain, for example, the rise in cases since 2003 may be due to the legislative reform that took place in that year when two articles of the Spanish Penal Code referring to abuse and habitual abuse were modified [2]. This legal amendment deemed abuse more serious, so family violence behaviors previously considered misdemeanors became felony crimes.

Ascertaining the true prevalence of APV is challenging. Research estimates suggest that between 5 and 21% of young people in the community have reportedly physically abused their parents [3]. However, these abuse estimates vary depending on the type of violence considered (verbal, psychological, emotional, economic, or physical) and the measure used. In some studies, this prevalence rises to 90% or more when considering verbal abuse [4]. 

The measurement of APV in research is complex. There are five commonly used questionnaires: The Parent-Child Conflict Tactics Scales [5,6]; the Child-to-parent violence subscale from the Scale of Intra-Family Violence [7]; the Child-to-parent aggression questionnaire [4] and the Child-to-parent violence questionnaire [8,9]. These instruments generally assess a wide range of behaviors, scoring the frequency of each behavior without considering the severity of behaviors. As such, individuals who engage in frequent, low-level aggressive behavior (e.g., yelling at parents) could receive higher scores from these instruments than individuals who engage in infrequent severe behaviors (e.g., physical assault resulting in criminal charges). There is no consensus on which behaviors or combination of behaviors constitute APV or how frequently they need to occur for them to be considered abusive.

Commonly in research, the presence of a single behavior is used as an indicator of APV, or researchers select cut-off points with limited empirical justification [10]. For example, the authors of the CPAQ defined severe physical aggression as instances in which a physical assault occurred at least 3–5 times in the last year. For severe psychological aggression, the behavior had to happen more than six times in the past year [4]. However, by that definition, adolescents would only have to yell at their parent(s) once every other month to meet the threshold for abuse. Considering that conflict with parents is somewhat normative during adolescence, it is unclear whether this threshold would discriminate APV from defiant or disrespectful behavior towards parents [11]. 

To address the complexity regarding the measurement of abuse, Simmons et al. [10] suggest considering the social and cultural context in which violence takes place since the interpretation of children’s behavior toward parents depends on social norms. They point out that fathers and mothers must set the threshold for abuse, as they are the potential victims of this type of violence. To assess this threshold, they developed the Beliefs About Child-to-Parent Abuse Questionnaire (BACPAQ) to investigate which behaviors towards parents have the potential to be considered abusive and at what point these behaviors deviated enough from the social norms to be perceived as abusive. In their study, participants considered that a physical assault only had to occur once to be considered abusive. In contrast, psychologically abusive behaviors had to occur more frequently to be perceived in the same way. 

Drawing upon the research investigating the thresholds of abuse using the BACPAQ, Simmons et al. [12] developed the Abusive Behavior by Children-Indices (ABC-I). The ABC-I was designed to provide an evidence-based threshold for APV that incorporates both frequency and severity of abuse and differentiates between abusive and non-abusive behavior patterns based on parents’ norms about abuse. The ABC-I was developed as an index because APV is a formative construct composed of observable phenomena that do not exist in their absence. Scales traditionally used to measure APV are based on Classical Test Theory, assuming that abuse is a reflective construct that underlies observable phenomena and that it exists without their presence. Using an index instead of a scale implies the consideration that APV is a behavior, not a trait [12].

BACPAQ and ABC-I have so far only been used with an Australian sample. Therefore, the results may not be generalizable to other cultures; behaviors considered normal or abusive in one culture may not be perceived similarly in another. Holt [13] states that cultural norms may affect the commission of APV and the individual and social response to it in several ways: (1) “the attitudes about the nature of violence and its acceptability, within and outside the family”; (2) “the extent to which family relations are […] a ‘private matter’”; (3) “the nature of family formations” (p. 851); and (4) “the position of children in relation to their parents within a culture, including the rights of children and the legal responsibilities of parents in relation to them”, as well as “the position and rights of women and men within a culture, including the role of mothers and fathers” (p. 850).

Besides the role of mothers and fathers, the roles of sons and daughters are also gender sensitive. Perpetrators’ gender has been addressed in APV research to analyze differences in the amount and type of violence exerted by boys and girls [14]. Some studies have also explored the differential characteristics of boys and girls who are violent with their parents, both in judicial [15] and in community samples [16,17,18]. However, it has not yet been studied whether the gender of the perpetrator affects APV’s social perception. That is, whether the same conduct may be considered more serious when perpetrated by a son (son-to-parent violence) rather than a daughter (daughter-to-parent violence) or vice-versa. Research on adult intimate partner violence has shown that abuse is perceived as more severe when perpetrated by a man [19]. In the study by Wilson and Smirles [19], for example, the differences in the participants’ perceptions of the severity of abuse, depending on whether it was perpetrated by a man or a woman, were interpreted as considering the consequences for the victim to be more severe in the first case. Underlying this reasoning is the stereotype that men have more strength and are larger in size than women. In addition to stereotypes, gender roles and differential socialization according to gender place men and women in different positions within society, with expectations of dominance for men and submission for women. Thus, a son engaging in abusive behavior towards his parents might be perceived as more dangerous and have more potential to cause harm than a daughter, and thus be judged more severely.

Therefore, the aim of this study is to explore the normative beliefs about APV related to the perpetrator’s gender in a sample of Spanish parents, by developing the Spanish adaptations of the BACPAQ and the ABC-I. With this purpose, two studies were carried out. In the first, BACPAQ was translated and administrated to a sample of Spanish parents, assessing differences in social perception between son-to-parent and daughter-to-parent violence. We expected that parents would judge the behaviors of sons more harshly than that of daughters. In the second study, we developed a Spanish adaptation of the ABC-I, using the Spanish parent’s scores and thresholds for abuse from the Spanish adaptation of the BACPAQ.

## 2. Study 1: Differences in Normative Beliefs between Son-to-Parent and Daughter-to-Parent Violence

### 2.1. Materials and Methods

#### 2.1.1. Participants

The sample included 329 Spanish parents (253 mothers and 76 fathers) aged 19 to 81 (*M* = 46.32, *SD* = 10.11). The sample’s offspring were aged 1 to 60 years. The educational levels were primary studies (2%), compulsory education (13.9%), mid-level professional training (5.8%), high school level (32.8%), and university studies (42.5%).

#### 2.1.2. Instruments

The Beliefs About Child-to-Parent Abuse Questionnaire (BACPAQ) [10] comprises 40 items representing behaviors considered APV in previous literature. Participants read the following instructions: “You are asked to provide your views/perceptions about conflict between a child and a parent. Below is a list of behaviors. For each behavior, please rate hypothetically how often would the behavior have to occur for it to be considered abusive towards a parent”. They are asked to answer using a 7-point Likert-type scale with the labels: once (1), few times (2), monthly (3), weekly (4), daily (5), several times a day (6), and it is not abusive (7). This last option was made available for those who considered that the behavior was not a form of abuse regardless of its frequency. A Spanish adaptation of the BACPAQ was developed and administered in this study (available from the corresponding author upon request). 

#### 2.1.3. Procedure

The BACPAQ adaptation to Spanish was carried out using the procedure described by Muñiz et al. [20] in their guidelines for the translation and adaptation of instruments. The preservation of the item’s meaning was prioritized over the literal translation. A pilot study was then conducted with a small sample (*n* = 36) to check for comprehension of the instructions and item content and format errors. As participants in this pilot study had difficulties with the response scale, the following example was added to illustrate how to choose a response: “If you think that Shouting and yelling is not abuse regardless of how often it occurs, choose 7. If you think that Shouting and yelling is abuse as soon as it occurs, even if it has only happened once, choose 1. If you think that Shouting and yelling start to be abusive when it happens several times a day, choose 6.” 

The questionnaire was accessed online through a link distributed by university students using the snowball sampling technique. Students were asked to find, in their immediate surroundings and social networks, fathers and mothers willing to collaborate by filling out the questionnaire. In the instructions, participants were randomly told that the violence was carried out by either a daughter or a son. They voluntarily completed the survey after being informed that the research focused on conflict between parents and their offspring. Anonymity and confidentiality of their responses were assured, and before accessing the questionnaire items, they gave their informed consent. Items’ presentation order was randomized to control the carry-over effect. This study was conducted in accordance with the Declaration of Helsinki and approved by the Ethics Committee of the Universidad de La Laguna (CEIBA2022-3224).

#### 2.1.4. Data Analyses 

Data analyses were carried out using the SPSS v.22.0 statistical package. The frequency at which parents considered that the 40 behaviors on the tool crossed the threshold from normative to abusive was analyzed. A cut-off point was obtained for each BACPAQ behavior using the 80th percentile as the consensus, regardless of the response distribution, to reflect the opinions of 80% of the participants about the frequency with which the behavior must occur to be considered a form of abuse [21]. The cut-off points for the two conditions—son or daughter as perpetrator—were calculated separately. The differences between the thresholds at which behaviors were identified as abusive in both conditions were analyzed using Chi^2^ tests of independence, and the probability that parents would identify a behavior as abusive, depending on the aggressor’s gender with Odds ratios.

### 2.2. Results

The results of the analysis described above are shown in Table 1.

Two behaviors were not considered abusive, regardless of the child gender: Rolled eyes at a parent and Talked back to parent. Fourteen of the remaining thirty-eight behaviors had thresholds for abuse that were different when perpetrated by a son and by a daughter, but the differences were only statistically significant for three behaviors: Became upset because chores were not done how or when he/she wanted them to be done (χ^2^(1; *n* = 329) = 5.45, *p* = 0.020, *OR* = 0.67, 95% *CI* = 0.10–1.24), Threatened to break or smash objects in the house (χ^2^(1; *n* = 329) = 5.25, *p* = 0.022, *OR* = 0.69, 95% *CI* = 0.09–1.29), and Stole parent’s money or possessions (χ^2^(1; *n* = 327) = 4.77, *p* = 0.029, *OR* = 0.64, 95% *CI* = 0.06–1.22). In all cases, the thresholds were lower for sons than for daughters. This means that parents reported that for these behaviors to be considered abuse when perpetrated by a daughter, they had to occur more frequently than when perpetrated by a son. The ORs suggested that parents were approximately six times more likely to identify these behaviors as abusive if they were perpetrated by a son than by a daughter.

## 3. Study 2: The Spanish Adaptation of the ABC-I

### 3.1. Materials and Methods

#### 3.1.1. Participants

We selected a subsample of participants from Study 1 to ensure that the Spanish adaptation of the ABC-I was developed with participants who met the same requirements as those in the original study [12]). This subsample was composed of 158 mothers and 48 fathers aged between 30 and 73 (*M* = 49.20, *SD* = 7.01), with children aged between 13 and 25 years. 

#### 3.1.2. Instruments

The Beliefs About Child-to-Parent Abuse Questionnaire (BACPAQ) [10] was used in its adaptation to Spanish, as described in Study 1.

#### 3.1.3. Procedure

The instructions given to the participants and the sample contacting process were the same as those described in Study 1. This study was also conducted in accordance with the Declaration of Helsinki and approved by the Ethics Committee of the Universidad de La Laguna (CEIBA2022-3224).

#### 3.1.4. Data Analyses

Data analysis was carried out using the SPSS v.22.0 statistical package. The criteria for consensus was established by setting the 80th percentile as the cut-off point for each of the BACPAQ behaviors [21], as described in Study 1, irrespective of the perpetrator’s gender. To develop the Spanish adaptation of the ABC-I, we followed the procedure described by Simmons et al. [12]. Spearman’s correlations and Variance Inflation Factors (VIF) were calculated to assess the collinearity of the BACPAQ’s items. As ABC-I is an index and not a scale, items with null or weak inter-correlations were retained as they were seen to capture a unique characteristic of APV [22]. Items were removed or collapsed if they were highly correlated (i.e., ρ > 0.64, VIF > 10; Tolerance < 0.1) [23]. If items with differing thresholds for abuse were collapsed, the threshold of the item with the greater severity was applied.

### 3.2. Results

#### 3.2.1. The Threshold for Abuse for Spanish Parents with Adolescent Children, Irrespective of the Perpetrator’s Gender

The frequency thresholds for abuse for the BACPAQ behaviors, calculated irrespective of the perpetrator’s gender, are shown in Table 2. Two of the 40 behaviors were not considered abusive, as in the original study: Rolling eyes at a parent and Talking back to a parent were not considered abusive behaviors regardless of their frequency. The behaviors Becoming upset because chores were not done how or when they wanted them to be done and Blaming parent for child’s own behavior were perceived as abusive if they were repeated several times a day and daily, respectively, whereas in the original study, they were not considered abuse at all. Behaviors regarding physical violence were rated as abusive even if they happened once. Verbally, psychologically, and economically violent behaviors had lower thresholds as they increased in severity, ranging from several times a day (e.g., Shouted or yelled) to a few times (e.g., Stole parent’s money or possessions).

For illustrative purposes, Table 2 indicates the sixteen behaviors for which the 80th percentile frequency threshold for abuse differed in our sample to that in the study of Simmons et al. [12]. The threshold was lower for nine behaviors, meaning that, for Spanish parents, these behaviors needed to happen fewer times to be considered abusive: Swearing at a parent, Trying to keep parent from doing something that he/she wanted to do, Purposefully making parent feel guilty so that the parent would do what he/she wanted, Swearing at a parent in front of others, Slamming or kicking objects in the house, Insulting or humiliating a parent and Threatening to break or smash objects in the house, Breaking or smashing objects in the house, and Purposefully collecting debt that parent had to pay. In contrast, the thresholds were higher for five behaviors, meaning that, for Spanish parents, these behaviors needed to happen more times to be considered abusive: Threatening to turn friends or family against parent, Making parent do something humiliating, Using pressure, exploitation, or threats to obtain money, Throwing something at parents without causing injury, and Grabbing or Pushing parent without causing injuries. 

#### 3.2.2. Developing the Spanish Adaptation of the ABC-I

To develop the Spanish adaptation of the ABC-I, Spearman’s correlations and Variance Inflation Factors (VIF) were calculated using the scores from the Spanish adaptation of the BACPAQ of the Spanish parents with adolescent children. All correlations were significant, ranging from *p* = 0.25 to 0.97. Items 22 to 40, all related to physical violence, were collapsed because of multi-collinearity (VIF > 10; Tolerance < 0.1) (items 22, 23, 25, 26, 29, 30, 31, 32, 33, 34, 35, 36, 37, 39, 40) or high correlations (*p* = 0.72–0.85; items 24, 27, 28, and 38). Likewise, three groups of items were combined due to correlations ranging from 0.64 to 0.71 (6, 9, 11 y 20; 10 and 13; 7, 8, 12, 15, 17, 18, 19, and 21). In the final Index, VIFs were below the cut-off of 10 (range = 1.41–9.33) and Tolerance was > 0.1 (range 0.11–0.71), confirming the absence of multi-collinearity. If items with differing thresholds for abuse were collapsed, the threshold of the item with the greatest severity was applied. The final tool was composed of nine items that can be added together to reach a total score or separated scores for verbal, economic, psychological, and physical violence (see Appendix A).

There is a total agreement in perceiving the different forms of physical violence as closely related and equally serious, and considering them as abuse from the first time they occur. There is also an agreement in seeing stealing money or possessions as a form of abuse when it occurs a few times. However, there are some differences in relation to verbal, psychological, and economic violence. Due to these differences, the behaviors had to collapse differently than in the original tool, as for items such as swearing with insulting (item 4), instead with shouting (item 3), and for those related to psychological violence involving control (item 8). There are also violent behaviors that constitute independent items that were not in the original ABC-I, such as items 1, 2, or 6.

The scoring of the Spanish adaptation of the ABC-I is the same as that of the original instrument, as detailed by Simmons et al. [12]. The scoring procedure for each item is different, depending on parents’ perceptions of how often the behavior described in the specific item would have to occur to be abusive. For each ABC-I item, a score of 16 is given to the threshold of abuse established using the responses to the BACPAQ. For example, in the Spanish sample, shouting or yelling at a parent had to occur daily, whereas punching a parent only had to occur once to be considered abusive and, therefore, the score of 16 is assigned to daily in the former case and to once in the latter. If a behavior was reported as occurring more or less frequently than its threshold for abuse, scores were increased or decreased by multiplying of dividing by two, respectively. A score of 16 was selected for the threshold because it was the lowest number that would result in a whole number if divided by a factor of 2 repeatedly. In this study, the possible scores for shouting or yelling at a parent ranged from 1 (once) to 16 (daily), while scores for punching a parent ranged from 16 (once) to 256 (daily). The total score is calculated by summing the scores of the nine items of the index. In addition, it is possible to obtain a score for psychological violence by summing items 1, 2, and 8; for verbal violence with items 3 and 4; for physical violence with items 5 and 9; and for economic violence with items 6 and 7 (see Appendix A). 

## 4. Discussion

This study aimed to explore the normative beliefs on APV related to the perpetrator’s gender in a sample of Spanish parents, by developing Spanish adaptations of the BACPAQ and the ABC-I. With this purpose in mind, two studies were carried out. First, the BACPAQ was translated and administrated to a sample of Spanish parents, assessing differences in social perception between son-to-parent and daughter-to-parent violence. In the second study, abuse thresholds were calculated irrespective of the perpetrator’s gender and used to develop a Spanish adaptation of the ABC-I. In this study, participants were parents who met the same requirements as the original study [12].

### 4.1. Differences in Abuse Threshold According to Gender

We expected that parents would judge sons’ behaviors more harshly than daughters’ (i.e., sons would need to carry out a behavior less often before it met the threshold for abuse). In line with expectations, sons were judged more harshly than daughters for all behaviors in which the cut-off points differed. However, these differences were only statistically significant for three behaviors referring to psychological violence. These results are consistent with the existing adult intimate partner violence (IPV) literature which suggests that psychologically aggressive behavior by men is judged more harshly than by women [24,25]. However, it is worth noting that the differences found were not very large, as thresholds for abuse did not differ according to perpetrator gender for most behaviors.

Concerning physical aggression, the thresholds suggest that any severity would be equally considered and generally not be tolerated by parents regardless of the gender of their child. These results were unexpected because in the adult IPV literature, aggressive or violent behavior by men is generally perceived as more harmful and less acceptable [25,26,27]. The reasons underlying this gender bias are that, according to gender stereotypes, men have more strength and are larger in size, with their behaviors having more serious consequences [19]. However, the results of this study make sense if we consider that they state at which point a behavior becomes abusive, whereas research on adult IPV focuses on perceived harm, fear, or acceptability according to the interaction between victim and perpetrator gender [25,26,27]. In the current study the victim was always a woman, the mother, and what varied was the perpetrator’s gender. This applies also to the scarce difference found in the thresholds for psychological and other types of abuse. It would be interesting for future research to assess the extent to which the gender of the perpetrator interacts with the gender of the victim when assessing normative beliefs about APV, and whether the dynamics vary depending on the type of abuse (physical, psychological, etc.).

### 4.2. The Spanish Adaptation of the ABC-I

Study 2 showed that Spanish and Australian parents agreed on the abusive nature of most behaviors in the BACPAQ, especially regarding thresholds for physically aggressive behaviors. Differences were found for sixteen behaviors, eleven of them referring to verbal, psychological, and economic violence, for which Spanish parents were more stringent, and five referring to behaviors, two including physical violence, for which Spanish parents were more lenient. 

In general terms, Spanish parents were stricter than Australian parents with children who break things at home and who insult or humiliate them. They also considered equally intolerable any form of threatening, intimidating, or manipulative behavior, except threatening them with an object, which was seen as physical aggression. In the same vein, two forms of psychological abuse, becoming upset with parents because chores were not done how or when wanted and blaming parents for their own behavior, were not abusive for Australian parents, but were for Spanish parents when they occurred daily. Lastly, Spanish parents evaluated purposefully collecting debt that parents had to pay as something different from other forms of abuse but as serious as stealing and threatening behaviors. These differences could make sense if we consider that some digital media have lately been portraying Spanish parents as the most protective and controlling when compared to those of other countries in Europe, the Middle East, and North Africa [28]. However, it seems unreasonable to think that differences in parents’ normative beliefs about APV can be explained exclusively in terms of the parenting styles in fashion in a country at a given time. These preferences may reflect underlying political and economic conditions that have contributed, among other effects with psychological consequences, to delayed childbearing, which in turn has produced widening age gaps between parents and children. Therefore, future research on cultural differences in parenting style would help us to better understand normative beliefs on APV.

Spanish parents were more lenient for five abusive behaviors: two psychological, one economic, and two physical. The two psychically violent behaviors were grabbing or pushing the parent without causing injuries and throwing something at the parent without causing injuries. These differences may be due to Spanish culture, in which physical contact and, apparently, violent games without harm are common between parents and their offspring. In this sense, Calvete et al. [29] have described the family cultural context in Spain as being characterized by horizontal relationships, with adolescents spending more time at home and maintaining high degrees of family interrelation, solidarity, and dependence. Young people in Spain become independent from their parents in a home of their own later than other young people in the EU (28.9 vs. 26.5 years old on average [30]). Spain and Australia are countries with different languages, history, and legal systems, as well as religious and legal (common/continental law) traditions. These differences may explain why Spanish parents are more stringent than Australian parents towards 27.5% of the violent behaviors, including the two behaviors considered not abusive in the Australian sample. To further understand what might account for the differences in the cut-off points in the two populations, a more in-depth analysis of the cultural and relationship contexts and dynamics is needed.

Regarding the original ABC-I and its Spanish adaptation, there is total agreement in perceiving the different forms of physical violence and the behavior of stealing money or possessions. This is not surprising since the norms of both Australian and Spanish parents resemble just what criminal law in many countries states: offenses that physically harm people are the most serious, followed by offenses against possessions. However, there are differences that reflect parents’ normative beliefs about APV in both cultures that involve verbal, psychological, and economic violence. It is worth noting that all forms of control collapsed in one item, that some behaviors are associated differently (e.g., swearing with insulting instead of shouting), or that there are independent behaviors that did not appear in the original tool (e.g., blaming parents). These differences make it difficult to conduct transcultural studies that allow empirical comparisons using the same instrument. Future research should delve deeper into how to solve this difficulty; maybe focusing more on specific APV categories of behaviors or specific behaviors [16], as provided by the BACPAQ, than in global measures as the one resulted from the ABC-I and other available APV questionnaires. This is especially important for research that conceptualizes APV as behaviors and not as a trait.

### 4.3. Limitations and Future Directions

The main limitation that Studies 1 and 2 share is the disproportion between participants’ genders. This imbalance is habitual in responses to online pools, in which women usually answer more than men [31]. Still, it could be reduced in future research by collecting data with trained interviewers or on payment platforms that allow you to select filters to collect data. Mothers are indeed victims of APV more often than fathers [13]. However, fathers’ normative beliefs about APV may play an important role in the origin and maintenance of this type of violence and should be analyzed. Further, fathers may have different perceptions than mothers about what constitutes abuse, particularly when considering differences between sons and daughters. For instance, research in adult IPV literature suggests that men are more likely to view psychologically aggressive behavior as acceptable [32]. As such, samples with larger proportions of men may yield different results as father’s perceptions of their children’s behavior may differ from mother’s.

A second limitation of Study 2 is that scores in the Spanish adaptation of the ABC-I were not compared with those of other instruments used to measure APV in Spain, such as the Child-to-parent aggression questionnaire [4] or the Child-to-parent violence questionnaire [8,9]. Future research should do this using a larger and more balanced sample. In addition, it would be interesting to analyze whether the relationships between APV and risk factor found in research using these other tools are replicated in the Spanish adaptation of the ABC-I. This type of research would provide evidence of validity both for the ABC-I and for the other two instruments. 

A third limitation of this study is that parents were asked to hypothetically consider how frequently a behavior had to occur for it to constitute abuse. However, we were unable to ascertain what information participants were relying on to make these judgments. This limitation has two implications for future research. First, although normative beliefs will be similar for people belonging to the same community, it would be interesting to assess differences in the introjection of these norms between parents who have been victims of APV and those who have not. This evidence would be useful for the diagnoses of APV cases by establishing the area under the curve (AUC) to determine whether the new instrument discriminates between parents who report that their child’s behavior is abusive and those who do not [12]. Second, resembling research on perceptions of adult IPV, it may be useful to use experimental designs, presenting cases in which variables of interest have been manipulated to investigate how victim, perpetrator, or behavior characteristics affect perceptions of APV. The use of experimental research designs would be helpful to better ascertain what informs parents’ judgments about whether a behavior carried out by their son or daughter is considered abusive.

## 5. Conclusions

Despite its limitations, this research provides valuable insights into parents’ normative beliefs about APV and the impact of gender and culture on these standards. Indeed, to the best of our knowledge, this is the first study that empirically contrasts parents’ normative beliefs about APV perpetrated by a son or by a daughter and explores cultural differences between an Anglo-Saxon and a Latin country in those beliefs. The results show an agreement on the abusive nature of most physical APV, regardless of the perpetrator’s gender and the culture in which violence occurs. Cultural differences seem to influence the thresholds that allow us to identify verbal, psychological, and economical violence. The adaptation to Spanish of the BACPAQ, and especially of the ABC-I, may help to better differentiate cases that constitute abuse from those which display disrespectful or defiant behaviors according to cultural norms. These evidence-based tools, which incorporate both frequency and severity of behaviors, can be useful to better enable identification of and intervention in cases of APV. The main practical implication of using tools like these in research, as well as in clinical and forensic settings, would be to take a further step in depathologizing some cases of APV that are nothing more than defiant or disrespectful behavior. In this way, legal and clinical intervention resources would be able to concentrate on the most severe cases, while educative resources would focus on improving parent–child relationships in general terms. Psychological research on both types of negative adolescent behaviors toward parents would support both intervention approaches. 

## Figures and Tables

**Table 1:** Cut-off Points for the 40 Potentially Abusive Behaviors in the BACPAQ Depending on the Perpetrators’ Gender.

		Frequency Cut-off Point for Abuse		
Behavior		Son (*n* = 166)	Daughter (*n* = 163)	Odds Ratio
1	Rolled eyes at a parent	Not abusive	Not abusive	
2	Talked back to the parent	Not abusive	Not abusive	
3	Became upset because chores were not done how or when he/she wanted them to be done	Daily	Several times *	0.67
4	Blamed parent for child’s own behavior	Weekly	Several times ^ns^	
5	Shouted or yelled	Daily	Several times ^ns^	
6	Swore at parent	Weekly	Weekly	
7	Tried to keep parent from doing something that he/she wanted to do	Weekly	Daily ^ns^	
8	Purposefully made parent feel guilty so that the parent would do what he/she wanted	Monthly	Weekly ^ns^	
9	Swore at parent in front of others	Monthly	Monthly	
10	Slammed or kicked objects in the house	Few times	Monthly ^ns^	
11	Insulted or humiliated parent	Few times	Few times	
12	Threatened to break or smash objects in the house	Few times	Monthly *	0.69
13	Broke or smashed objects in the house	Few times	Few times	
14	Purposefully collected debt that parent had to pay	Few times	Monthly ^ns^	
15	Attempted to intimidate parent	Few times	Few times	
16	Stole parent’s money or possessions	Few times	Monthly *	0.64
17	Threatened to hurt him/herself or others if parent did not do what he/she wanted	Few times	Few times	
18	Threatened to turn friends or family against parent	Monthly	Monthly	
19	Threatened to burn parent’s possessions	Once	Few times ^ns^	
20	Made parent do something humiliating	Once	Few times ^ns^	
21	Used pressure, exploitation, or threats to obtain money	Few times	Few times	
22	Threatened parent with an object	Once	Once	
23	Threw something at parent—no injury	Few times	Monthly ^ns^	
24	Grabbed or pushed parent—no injury	Few times	Few times	
25	Hit or slapped parent—no injury	Once	Once	
26	Kicked or punched parent—no injury	Once	Few times ^ns^	
27	Threw something at parent—minor injury	Once	Once	
28	Grabbed or pushed parent—minor injury	Once	Few times ^ns^	
29	Hit or slapped parent—minor injury	Once	Once	
30	Kicked or punched parent—minor injury	Once	Once	
31	Threw something at parent—major injury	Once	Once	
32	Grabbed or pushed parent—major injury	Once	Once	
33	Hit or slapped parent—major injury	Once	Once	
34	Kicked or punched parent—major injury	Once	Once	
35	Used a weapon against parent	Once	Once	
36	Burned or scalded parent	Once	Once	
37	Choked parent	Once	Once	
38	Kept parent from getting medical care	Once	Once	
39	Forcibly confined parent	Once	Few times ^ns^	
40	Burned parent’s possessions	Once	Once	

Note: * *p* < 0.05; ^ns^ *p* > 0.05.

**Table 2:** Cut-off Points for the 40 Potentially Abusive APV Behaviors in the Spanish Adaptation of the BACPAQ.

Behavior		Frequency Threshold for Abuse
1	Rolled eyes at a parent	Not Abusive
2	Talked back to the parent	Not Abusive
3	Became upset because chores were not done how or when he/she wanted them to be done	Several times a day *
4	Blamed parent for child’s own behavior	Daily *
5	Shouted or yelled	Several times a day
6	Swore at parent	Weekly *
7	Tried to keep parent from doing something that he/she wanted to do	Weekly *
8	Purposefully made parent feel guilty so that the parent would do what he/she wanted	Weekly *
9	Swore at parent in front of others	Monthly *
10	Slammed or kicked objects in the house	Monthly *
11	Insulted or humiliated parent	A few times *
12	Threatened to break or smash objects in the house	A few times *
13	Broke or smashed objects in the house	A few times *
14	Purposefully collected debt that parent had to pay	A few times *
15	Attempted to intimidate parent	A few times
16	Stole parent’s money or possessions	A few times
17	Threatened to hurt him/herself or others if parent did not do what he/she wanted	A few times
18	Threatened to turn friends or family against parent	Monthly **
19	Threatened to burn parent’s possessions	A few times
20	Made parent do something humiliating	A few times **
21	Used pressure, exploitation, or threats to obtain money	A few times **
22	Threatened parent with an object	Once
23	Threw something at parent—no injury	A few times **
24	Grabbed or pushed parent—no injury	A few times **
25	Hit or slapped parent—no injury	Once
26	Kicked or punched parent—no injury	Once
27	Threw something at parent—minor injury	Once
28	Grabbed or pushed parent—minor injury	Once
29	Hit or slapped parent—minor injury	Once
30	Kicked or punched parent—minor injury	Once
31	Threw something at parent—major injury	Once
32	Grabbed or pushed parent—major injury	Once
33	Hit or slapped parent—major injury	Once
34	Kicked or punched parent—major injury	Once
35	Used a weapon against parent	Once
36	Burned or scalded parent	Once
37	Choked parent	Once
38	Kept parent from getting medical care	Once
39	Forcibly confined parent	Once
40	Burned parent’s possessions	Once

Note: * Spanish parents have a lower threshold than Australian ones. ** Spanish parents have a higher threshold than Australian ones.

## Data Availability

The raw data supporting the conclusions of this article will be made available by the authors without undue reservation.

## Appendix A

*Adaptación Española del ABC-I* *A continuación, encontrarás una lista de comportamientos. Por favor, indica si te has comportado de la siguiente forma con tu [madre/padre] en los últimos 12 meses.*							
		Nunca	Una vez	Varias veces	Mensual-mente	Semanal-mente	Diariamente
1.	Enfadarte con tu padre/madre porque las tareas de la casa no se han hecho cómo o cuándo querías.	0	1	2	4	8	16
2.	Echarle la culpa de tu comportamiento a tu madre/padre.	0	1	2	4	8	16
3.	Gritar o chillar a tu padre/madre.	0	1	2	4	8	16
4.	Insultar, maldecir o humillar a tu madre/padre.	0	8	16	32	64	128
5.	Romper, destrozar, golpear o patear objetos de la casa.	0	8	16	32	64	128
6.	Contraer a propósito una deuda que tuviera que pagar tu madre/padre.	0	8	16	32	64	128
7.	Robarle dinero o pertenencias a tu madre/padre.	0	8	16	32	64	128
8.	Amenazar, intimidar u obligar a tu madre/padre a hacer algo que no quiere o impedir que haga algo que quiere.	0	8	16	32	64	128
9.	Agredir físicamente a tu madre/padre (p.e., tirarle algo, agarrarle o empujarle, golpearle o abofetearle, darle patadas o puñetazos, etc.)	0	16	32	64	128	256
Por favor, marca todos los comportamientos físicamente agresivos contra tu (madre/padre) en los últimos 12 meses: □ le tiró algo a [él/ella/ellos] □ le empujó □ le agarró □ le golpeó o abofeteó □ le dio un puñetazo □ le dio una patada □ le estranguló □ utilizó un arma contra él/ella/ellos □ otro__________________________________ Por favor, marque si le causó alguna lesión a su/s [madre/padre/padres] en los últimos 12 meses: □ lesión leve (por ejemplo, cortes, moratones, etc.) □ lesión grave (por ejemplo, rotura de huesos o dientes, lesión en la cabeza, etc.)							
Procedimiento de corrección: Violencia verbal = 3 + 4. Violencia física = 5 + 9. Violencia psicológica = 1 + 2 + 8. Violencia económica = 6 + 7. Violencia total = 1 + 2 + 3 + 4 + 5 + 6 + 7 + 8 + 9.							

*Translation to English of the Spanish adaptation of the ABC-I* *Below is a list of behaviors. Please indicate whether you have behaved in the following ways towards your (father/mother) within the past 12 months].*							
		Never	Once	A few times	Monthly	Weekly	Daily
1.	Becoming upset with your (mother/father) because chores were not done how or when you wanted them to be done.	0	1	2	4	8	16
2.	Blaming your (mother/father) for your own behavior.	0	1	2	4	8	16
3.	Shouting or yelling at your (mother/father).	0	1	2	4	8	16
4.	Insulting, swearing, or humiliating your (mother/mother).	0	8	16	32	64	128
5.	Breaking, smashing, slamming, or kicking objects in the house.	0	8	16	32	64	128
6.	Purposefully collecting debt that your (father/father) had to pay.	0	8	16	32	64	128
7.	Stealing your (mother/father’s) money or possessions.	0	8	16	32	64	128
8.	Threatening, intimidating, or making your (mother/father) do something s/he did not want to do or preventing her/him from doing something s/he wanted to do.	0	8	16	32	64	128
9.	Acted physically aggressively towards your (mother/father) (e.g., threw something, grabbed or pushed, hit or slapped, kicked or punched, etc.).	0	16	32	64	128	256
Please check all physically aggressive behaviors that you used against your [parent/s] in the past 12 months: □ threw something at [her/him/them] □ pushed □ grabbed □hit or slapped □ punched □ kicked □ strangled □ used a weapon against her/him/them □ other ___________________________________________ Please check if you injured your [parent/s] in the past 12 months: □ minor injury (e.g., cuts, bruises, etc.) □ major injury (e.g., broken bones or teeth, head injury, etc.)							
Scoring procedure: Verbal violence = 3 + 4. Physical violence = 5 + 9. Psychological violence = 1 + 2 + 8. Economic violence = 6 + 7. Total Score = 1 + 2 + 3 + 4 + 5 + 6 + 7 + 8 + 9							
